# Dysregulated mRNA Translation in the G2019S LRRK2 and LRRK2 Knock-Out Mouse Brains

**DOI:** 10.1523/ENEURO.0310-21.2021

**Published:** 2021-11-30

**Authors:** Jungwoo Wren Kim, Xiling Yin, Ian Martin, Yulan Xiong, Stephen M. Eacker, Nicholas T. Ingolia, Ted M. Dawson, Valina L. Dawson

**Affiliations:** 1 Neuroregeneration and Stem Cell Programs, Institute for Cell Engineering, Johns Hopkins University School of Medicine, Baltimore, MD 21205; 2 Department of Physiology, Johns Hopkins University School of Medicine, Baltimore, MD 21205; 3 Department of Molecular and Cell Biology, University of California, Berkeley, Berkeley, CA 94720; 4 Department of Neurology, Johns Hopkins University School of Medicine, Baltimore, MD 21205; 5 Adrienne Helis Malvin Medical Research Foundation, New Orleans, LA 70130; 6 Solomon H. Snyder Department of Neuroscience, Johns Hopkins University School of Medicine, Baltimore, MD 21205; 7 Department of Pharmacology and Molecular Sciences, Johns Hopkins University School of Medicine, Baltimore, MD 21205; 8 Diana Helis Henry Medical Research Foundation, New Orleans, LA 70130

**Keywords:** LRRK2, RPS15, translation

## Abstract

The G2019S mutation in leucine-rich repeat kinase 2 (LRRK2) causes familial Parkinson’s disease (PD) and is also found in a subset of idiopathic cases. Prior studies in *Drosophila* and human induced pluripotent stem cell (iPSC)-derived dopamine neurons uncovered a pronounced effect of G2019S LRRK2 on mRNA translation. It was previously reported that G2019S LRRK2 promotes translation of mRNAs with complex 5′ untranslated region (UTR) secondary structure, resulting in increased expression of calcium channels and dysregulated calcium homeostasis in human dopamine neurons. Here, we show that dysregulated translation occurs in the brains of mammalian LRRK2 models *in vivo*. Through ribosome profiling studies of global translation, we observe that mRNAs with complex 5′UTR structure are also preferentially translated in the G2019S LRRK2-expressing mouse brain. Reporter assays suggest that this 5′UTR preference is independent of translation initiation factors. Conversely, translation of mRNAs with complex 5′UTR secondary structure is downregulated in LRRK2 knock-out (KO) mouse brain, indicating a robust link between LRRK2 kinase activity and translation of mRNA with complex 5′UTR structure. Further, substantia nigra pars compacta (SNpc) dopamine neurons in the G2019S LRRK2-expressing brain exhibit increased calcium influx, which is consistent with the previous report from human dopamine neurons. These results collectively suggest that LRRK2 plays a mechanistic role in translational regulation, and the G2019S mutation in LRRK2 causes translational defects leading to calcium dysregulation in the mammalian brain.

## Visual Abstract

**Figure:**
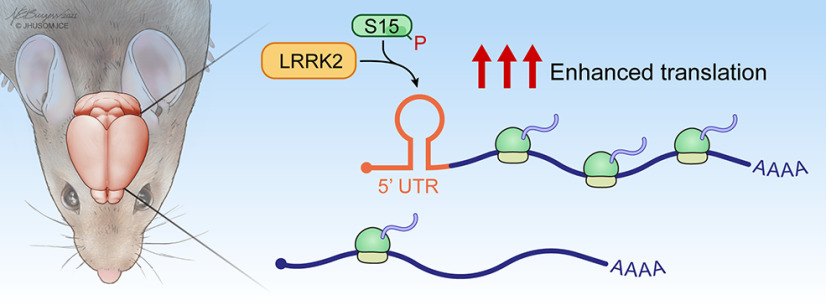

## Significance Statement

Parkinson’s disease (PD)-linked G2019S mutation of leucine-rich repeat kinase 2 (LRRK2) is known to cause abnormalities in mRNA translation. These translational defects were suggested to cause calcium dysregulation, thereby imposing a long-term cellular stress to dopamine neurons. While these effects of G2019S LRRK2 on mRNA translation have been seen in *Drosophila* brain tissues and cultured mammalian neurons, translational profiling of the mammalian brain expressing G2019S LRRK2 has not been reported. In this study, we employed ribosome profiling to survey mRNA translation in the brains of LRRK2 mouse models, thereby demonstrating that the G2019S LRRK2 mutation broadly alters mRNA translation in the mouse brain.

## Introduction

Dominant mutations in the leucine-rich repeat kinase 2 (LRRK2) gene are the most common genetic cause of familial Parkinson’s disease (PD), with the G2019S missense mutation being most frequent disease-causing mutation in LRRK2 (Martin et al., 2014a). The G2019S mutation enhances the kinase activity of LRRK2, leading to neurotoxicity (Greggio et al., 2006; Smith et al., 2006). While various cellular functions are associated with LRRK2 kinase activity, emerging evidence suggests that alterations in mRNA translation downstream of kinase activity plays an important role in PD pathogenesis (Imai et al., 2008; Gehrke et al., 2010; Martin et al., 2014b; Taymans et al., 2015). G2019S LRRK2 was reported to increase global protein synthesis through phosphorylation of the ribosomal protein S15 (uS19), and reduction of global protein synthesis is protective against G2019S LRRK2 neurotoxicity in a *Drosophila* model (Martin et al., 2014b). In addition, a recent study applying ribosome profiling to human dopamine neurons differentiated from patient-derived induced pluripotent stem cells (iPSCs) showed that the increased translation in G2019S LRRK2 leads to increased expression of genes responsible for calcium influx in neurons (Kim et al., 2020). While these studies presented potential mechanisms linking abnormal translation to cellular stress, the proposed mechanisms have yet to be tested in the mammalian brain.

## Materials and Methods

All animal protocols are in accordance with the regulations of Johns Hopkins University Animal Care and Use Committee and the National Institutes of Health *Guide for the Care and Use of Laboratory Animals.* Animals were housed in a 12/12 h light/dark cycle with free access to water and food. High-throughput sequencing data are available via NCBI GEO (accession number: GSE167704).

### Maintenance of LRRK2 transgenic mouse models

Generation and characterization of LRRK2 “Tet-off” transgenic mice and LRRK2 knock-out (KO) mice were previously reported (Andres-Mateos et al., 2009; Nikonova et al., 2012; Xiong et al., 2017). For transgenic mice, high copy number lines (569 line for GS, 763 line for GS/DA) were used (Xiong et al., 2017). Single transgenic mice Ca^2+^/calmodulin-dependent protein kinase II (CaMKII)-tTA or Tet-LRRK2) were used for breeding, and the breeding cages were maintained with doxycycline chow (Diet-Sterile, 200 mg/kg doxycycline, Bio-Serv) and fed *ad libitum*. Doxycycline food was switched back to regular food after weaning for transgene induction. Three- to four-month-old mice were used for ribosome profiling experiments (described below).

### Mouse primary cortical neuron culture

Dissipated primary cortical neurons were prepared from embryonic day (E)15 developing brain (CD1, Charles River or LRRK2 transgenic mice). Developing cortices were dissected in the dissecting medium (DMEM with 20% horse serum, 0.5 mm GlutaMax, and 6 μm glucose, Invitrogen), digested with TrypLE (Invitrogen), and plated at a concentration of 3 × 10^6^ cells for a plate. Culture plates were precoated with 15 μg/ml poly-L-ornithine. Cultures were maintained under Neurobasal (Invitrogen) medium with a serum-free supplement B-27 (Invitrogen) and 0.5 mm GlutaMax (Invitrogen).

### Immunocytochemistry of neurons

Cells were fixed with 4% paraformaldehyde for 15 min at room temperature, then permeabilized with 0.03% Triton X-100 for 15 min. The cells were washed then blocked for 1 h with 10% goat serum in PBS. The blocked cells were subsequently incubated with primary antibody for overnight at 4°C. On the following day, the cells were incubated with secondary antibody for 1 h at room temperature in a light controlled condition. After 3× wash with PBS buffer, the cells were mounted on cover slides with mounting media containing DAPI. All images were taken for analysis with Zeiss AxioObserver Z1 or LSM710 (Carl Zeiss) confocal laser scanning microscope under 20× or 40× oil objectives. Blinding was not performed with immunocytochemistry experiments. The following primary antibodies were used for immunocytochemistry: α-tyrosine hydroxylase (TH; 1:1000, EMD Millipore AB152).

### Ribosome profiling library generation

Ribosome footprinting and RNA-seq libraries were prepared by following a published protocol with several modifications made for mouse brain tissue (Ingolia et al., 2012).

#### Mouse brain

Brains of three- to four-month-old mice were dissected in TBS buffer with 100 μg/ml cycloheximide and immediately frozen in dry ice. Caudate putamen tissues from three mice of mixed gender (1:2 or 2:1 male:female ratio) were pooled; 2.5% of total lysate was subjected to Western blotting to ensure sufficient expression of transgene. The collected samples were homogenized in lysis buffer [10 mm Tris pH 7.5, 150 mm NaCl, 5 mm MgCl_2_, 0.5 mm DTT, 100 μg/ml cycloheximide, EDTA-free protease inhibitor (Roche), and 40 U/ml murine RNase Inhibitor (NEB)] with 12 strokes of high-speed motorized homogenizer (Glas-Col GT series) at 40% power. The lysates were briefly centrifuged for 10 min at 2000 × *g*. The supernatant was transferred to a new tube, added NP-40-1% final concentration, incubated 5 min on ice. The samples were centrifuged again for 10 min at 20,000 × *g*. The lysates were incubated in ice for 15 min, and centrifuged for 10 min at 20,000 × *g*. Total RNA concentration of lysate was measured by Qubit RNA BR Assay (Life Technologies), and the same amount of RNA was used across samples. The supernatant was split into two tubes for ribosome footprinting and RNA-seq library generation.

#### Ribosome footprinting

The lysates were treated with 15 μl of RNase I (Ambion) in 600-μl total reaction volume for 45 min at room temperature, and the reaction was stopped by adding 30 μl of SuperAse-In (Ambion). Sucrose cushion was performed with 1.7 g sucrose in 3.9 ml polysome buffer (10 mm Tris pH 7.5, 150 mm NaCl, 5 mm MgCl_2_, 0.5 mm DTT, 100 μg/ml cycloheximide, 20U/ml SuperAse-In), 4 h at 70,000rpm. The pellet was resuspended with 700 μl QIAzol (QIAGEN) reagent, incubated for 5 min at room temperature, 140 μl chloroform was added, vortexed for 15 s, and incubated again for 2 min at room temperature. The sample was centrifuged for 15 min at 12,000 × *g*, the 350 μl supernatant was mixed with 525 μl 100% EtOH. The mixture was loaded on an RNeasy Mini column (QIAGEN), and the RNA was extracted; 26–34 nt ribosome footprints were size-selected by Urea-PAGE, gel extraction and RNA purification. Ribo-Zero Gold kit (Illumina) was used for rRNA removal after the size selection. The rRNA depleted ribosome footprints were dephosphorylated by T4 polynucleotide kinase treatment, then Universal miRNA Cloning Linker (NEB) was added to the 3′ ends. Reverse transcription reaction was performed, and the cDNA was circularized by CircLigase II (Epicentre) reaction, and subjected to the PCR for final library generation.

#### RNA-seq

Total RNA was purified by a combination of QIAzol and RNeasy Mini as described. Ribo-Zero Gold kit was used for rRNA removal. RNA-seq library was generated from the total RNA by ScriptSeq v2 Library Preparation kit (Epicentre).

### Ribosome profiling data processing

Illumina HiSeq 2000 or 2500 were used for deep sequencing of the libraries. FASTX-Toolkit (http://hannonlab.cshl.edu/fastx_toolkit/) was used for the initial processing of the reads.

#### Ribosome footprinting libraries

Only adapter-containing reads were clipped. Reads shorter than 25 nt were discarded. The first nucleotide of the reads was trimmed. rRNA-mapped reads were discarded before genomic alignment.

#### RNA-seq libraries

Only adapter-containing reads were clipped, rRNA-mapped reads were discarded.

The processed reads were mapped to the UCSC genome database (mouse: mm9) by Tophat (2.0.11) with Bowtie2 (2.2.2). Maximum 1 mismatch was allowed for the alignments (for sequencing read counts, see Table 1).

**Table 1.** Sequencing read counts

Sample	Type	Mapped reads
Mouse control 1	Ribo	11,528,964
mRNA	35,167,826	
Mouse control 2	Ribo	14,236,436
mRNA	53,086,785	
Mouse control 3	Ribo	28,913,143
mRNA	37,446,599	
G2019S TG 1	Ribo	10,832,574
mRNA	37,329,310	
G2019S TG 2	Ribo	11,636,313
mRNA	42,813,645	
G2019S/D1994A TG 1	Ribo	11,391,779
mRNA	62,883,025	
G2019S/D1994A TG 2	Ribo	30,967,177
mRNA	33,887,256	
Mouse WT (vs KO) 1	Ribo	6,069,632
mRNA	44,668,380	
Mouse WT (vs KO) 2	Ribo	6,331,204
mRNA	8,025,070	
LRRK2 KO 1	Ribo	5,552,451
mRNA	65,571,861	
LRRK2 KO 2	Ribo	7,322,616
mRNA	18,455,674	
LRRK2 WT 3 (STR)	Ribo	22,256,190
LRRK2 WT 3 (VMB)	Ribo	8,773,494
LRRK2 KO 3 (STR)	Ribo	22,069,910
LRRK2 KO 3 (VMB)	Ribo	6,492,191

Ribo: ribosome profiling; mRNA: RNA-Seq; TG: transgenic mice; WT: wild type, KO: knock-out mice; STR: striatum; VMB: ventral midbrain.

### Internal ribosome entry site (IRES) reporter assays

pFR-HCV-xb, pFR-CrPV-xb vectors (from Phil Sharp Lab) were obtained from the Addgene depository (#11510 and #11509, respectively). The reporter vectors were co-transfected into CD1 wild-type mouse cortical neurons at day *in vitro* (DIV)5 with LRRK2-expressing or S15-expressing plasmids (or empty, respective expression plasmids for control) using Lipofectamine 2000 (Invitrogen) reagent. Luciferase to LRRK2/S15 expression vector ratio was 1:3. Culture medium was replaced (half-change) every 24 h to minimize any potential effects from the growth condition including starvation. Luciferase activity was measured at DIV7 by Dual-Glo Luciferase Assay System (Promega; for the IRES reporters) with Glomax 20/20 Luminometer (Promega). The lysates were subjected to the total RNA purification with DNase treatment for the transcript level measurement.

### Immunoblotting

Brain tissues were lysed with an automated homogenizer in RIPA buffer with 1% SDS [20 mm Tris-HCl (pH 7.5), 150 mm NaCl, 1 mm EDTA, 1% NP-40, 1% sodium deoxycholate, 1% SDS, protease inhibitors]. Lysates were incubated on a rotator for 1 h at 4°C, and spun down for 10 min × 12,000 × *g* at 4°C. Supernatant was collected, protein concentration was measured, and the lysate was mixed with 2× Laemmli sample buffer. Generation and characterization of rabbit polyclonal T136 phospho-S15 antibody was previously published (Martin et al., 2014b). Commercial antibodies: LRRK2: Neuromab (75-188, N138/6), P-eIF2ɑ (Cell Signaling Technologies, #9721), eIF2ɑ (Cell Signaling Technologies, #9722), and ATF4 (Millipore, ABE387).

### Ribosome profiling data analysis

Aligned reads were counted by either a Python package HTSeq (htseq-count) or an R package GenomicAlignments (summerizeOverlaps). Annotations and sequencing reads were handled using an R package GenomicFeatures. To avoid multiple counting on isoforms, transcript reference data were processed to have one unique annotation covering all isoforms (union of isoforms) per gene. Reads only in the CDS regions were counted. Transcripts with low read counts (<128 reads) were discarded. An R package DESeq (1.20) was used for calculating normalized expression from either ribosome footprinting or RNA-seq data based on a negative binomial distribution and generalized linear model. For the mouse data, replicates were initially analyzed independently to confirm reproducibility, and then analyzed in combination for the final analysis. For the human neuron data, biological triplicates were handled by DESeq. Translation efficiency (TE) was calculated based on the DESeq expression output. 5′ Untranslated region (UTR) estimated folding energy table was extracted from the UCSC genome database (fold5UTR field: mm9). For the 5′UTR estimated folding energy comparison, a control group with similar group size was randomly selected for each comparison to avoid potential bias from sample size differences. Transcript coordinates were calculated by a custom R script and re-aligned based on the rounded half point of the ribosome footprint [5′ end + (footprint length/2)]. For icSHAPE data analysis, icSHAPE (*in vivo*) results from mouse ES cells (GEO: GSE64169) were downloaded, converted to mm9 (UCSC liftOver), and merged with our mouse ribosome profiling data.

### Electrophysiological recordings

Mice (10–12 weeks old) were anesthetized and decapitated, and the brains were placed in ice-cold artificial CSF (ACSF) containing the following: 125 mm NaCl, 2.5 mm KCl, 1 mm MgSO_4_, 1.25 mm NaH_2_PO_4_, 26 mm NaHCO_3_, 2 mm CaCl_2_, and 10 mm D-glucose. Transverse brain slices containing substantia nigra pars compacta (SNpc; 350 μm) were prepared using a vibratome (Leica VT1200S). Sections were incubated in ACSF saturated with 95% O_2_ and 5% CO_2_, at 34°C for 60 min, and then at room temperature (22–24°C) until use. Recordings were performed at room temperature. All experiments were conducted in accordance with the National Institutes of Health *Guide for the Care and Use of Laboratory Animals*.

HEKA EPC10 amplifier (HEKA Elektronik) was used to perform electrophysiological recordings. For spontaneous and evoked action potentials (APs), a single slice was transferred into a submerged recording chamber and perfused constantly with oxygenated ACSF at a rate of 2 ml/min. DA neurons were visualized under a 40× water immersion objective by fluorescence and DIC optics (Carl Zeiss). The patch electrodes had a resistance of 2–5 MΩ and filled with solution containing the following: 126 mm K-gluconate, 8 mm KCl, 20 mm HEPES, 0.2 mm EGTA, 2 mm NaCl, 3 mm MgATP, 0.5 mm Na_3_GTP, and 0.05 mm Alexa Fluor 568 (pH 7.2, 290–300 mOsm/kg). Input resistance (Rin), series resistance (Rseries), and leak currents (Leak) were monitored throughout the experiment. Unstable recordings (>10% fluctuation of Rseries value) during the course of experiments were rejected for further analysis. Resting membrane potential was recorded in current clamp mode at 0 pA immediately after establishing whole-cell configuration. A series of hyperpolarizing and depolarizing step currents were injected to elicit APs. For whole-cell calcium currents, the external solution used contained 140 mm tetraethylammonium methanesulfonate (TEA-MeSO_3_), 10 mm HEPES, and 10 mm BaCl_2_ or CaCl_2_ (pH 7.4, 300–310 mOsmol/kg). The pipette solution contained 135 mm CsMeSO_3_, 5 mm CsCl, 1 mm MgCl_2_, 4 mm MgATP, 5 mm HEPES, and 5 mm EGTA (pH 7.3, 290–300 mOsmol/kg). Currents were recorded by holding the cell at –90 mV, before stepping to various potentials from –60 to +50 mV for 250-ms in 10-mV increments. Tetrodotoxin (TTX; 1 μm) was used to block voltage-gated sodium currents. Data were acquired by PatchMaster software (HEKA Elektronik), sampled at 10 kHz, and filtered at 2.9 kHz. APs and calcium currents were analyzed using Clampfit 10.5 software (Molecular Devices). Neurons labeled with Alexa Fluor 568 were confirmed by immunohistochemistry after recording.

## Results

**Figure 1.**
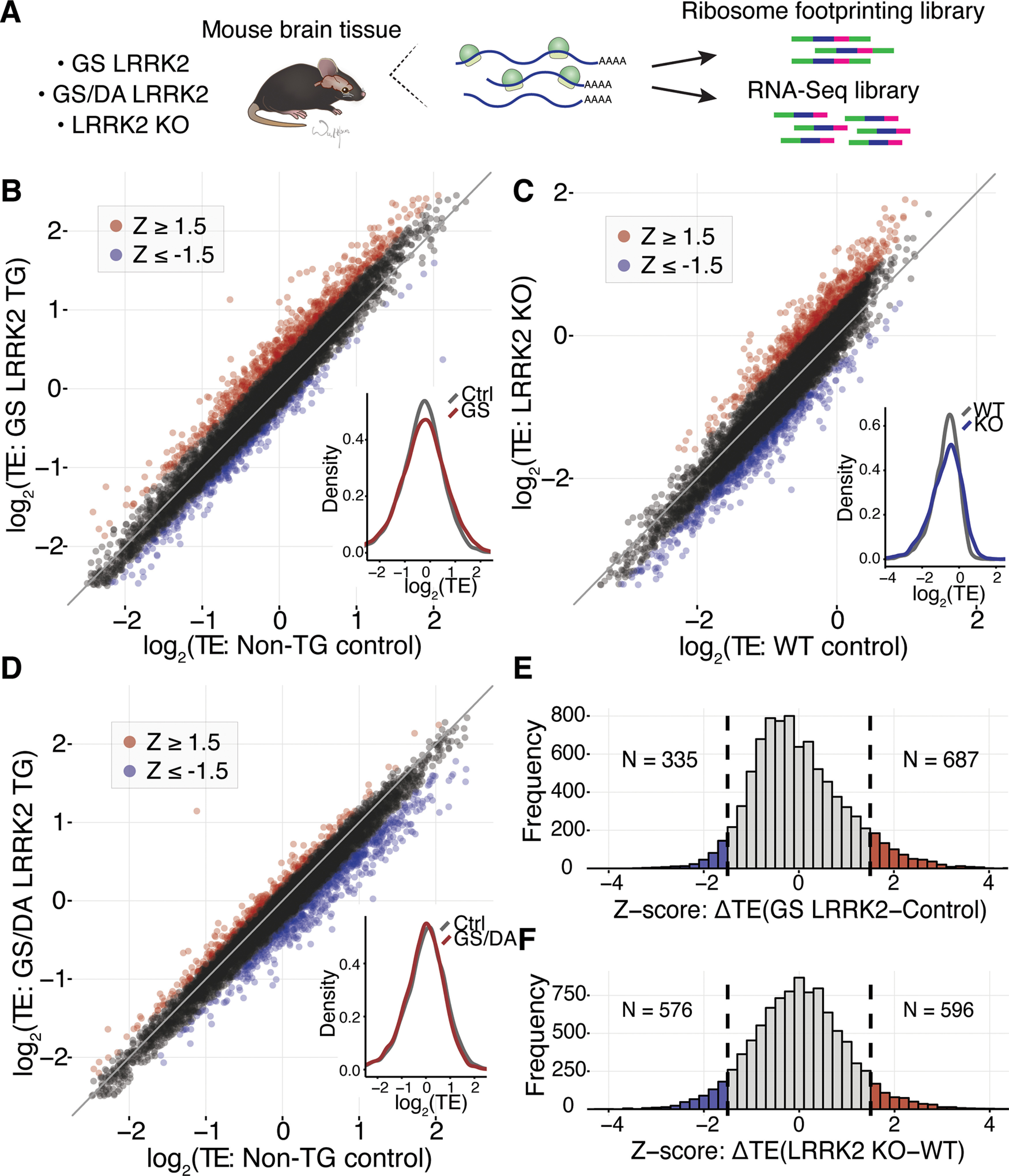
Broad alteration in mRNA translation in the G2019S LRRK2 mouse brain. ***A***, A schematic of ribosome profiling workflow with mouse brain tissue. ***B–D***, TE was calculated to estimate translational activity. Global TE distributions between (***B***) GS LRRK2 TG and non-TG control, (***C***) LRRK2 KO and WT, and (***D***) GS/DA LRRK2 TG and non-TG control were compared. All values are in log_2_, and each data point represents a single transcript. In scatterplots, centerline is a guideline with slope of 1, meaning that the dots on the line do not have TE value differences between the genotypes. SD of TE differences: 0.226 (GS LRRK2 vs control), 0.179 (GS/DA LRRK2 vs control), 0.273 (LRRK2 KO vs WT). Standard *z* score was calculated, and ±1.5 cutoff was used to select TE up and TE down genes. Triplet periodicity is normal across the results (Extended Data Fig. 1-1). ***E***, ***F***, Histogram of TE differences (δTE, ΔTE) between (***E***) GS LRRK2 TG and non-TG control or (***F***) LRRK2 KO and WT. *Z* score ±1.5 cutoff was used, and TE values are in log_2_. Each ribosome profiling experiment was firstly analyzed independently to ensure reproducibility. Two independent results were analyzed together by DESeq (Anders and Huber, 2010; *n* = 2). Expression analysis results including TE values were compiled (Extended Data Fig. 1-2).

We sought to characterize translational abnormalities in the brains of LRRK2 mouse models, focusing on the caudate putamen, where substantia nigra dopamine neurons project to and is linked to the pathology of PD. To obtain high expression of G2019S LRRK2 or kinase dead G2019S/D1994A LRRK2 transgenes, we crossed mice harboring doxycycline-regulated LRRK2 expression constructs with the CaMKII-tTA driver mice (Lee et al., 2013; Xiong et al., 2017). We then analyzed translation in the caudate putamen of the resulting G2019S LRRK2 or G2019S/D1994A LRRK2 transgenic mice, as well as LRRK2 KO animals (Fig. 1*A*; Extended Data Figs. 1-1, 1-2; Nikonova et al., 2012).

10.1523/ENEURO.0310-21.2021.f1-1 Extended Data Figure 1-1 Triple periodicity of ribosome profiling data. ***A***, ***B***, Triplet periodicity of ribosome profiling datasets were visualized to ensure the quality of the libraries. Transcript coordinates were re-aligned based on the rounded half point of the ribosome footprint [5′ end + (footprint length/2)]. Conserved triplet periodicity indicates that the libraries are faithfully representing translating ribosomes, ensuring the quality of the RPF libraries. There was no significant change found in ribosome footprint length, periodicity, and distribution in any LRRK2 mouse models (data not shown). Download Figure 1-1, TIF file.

**Figure 2.**
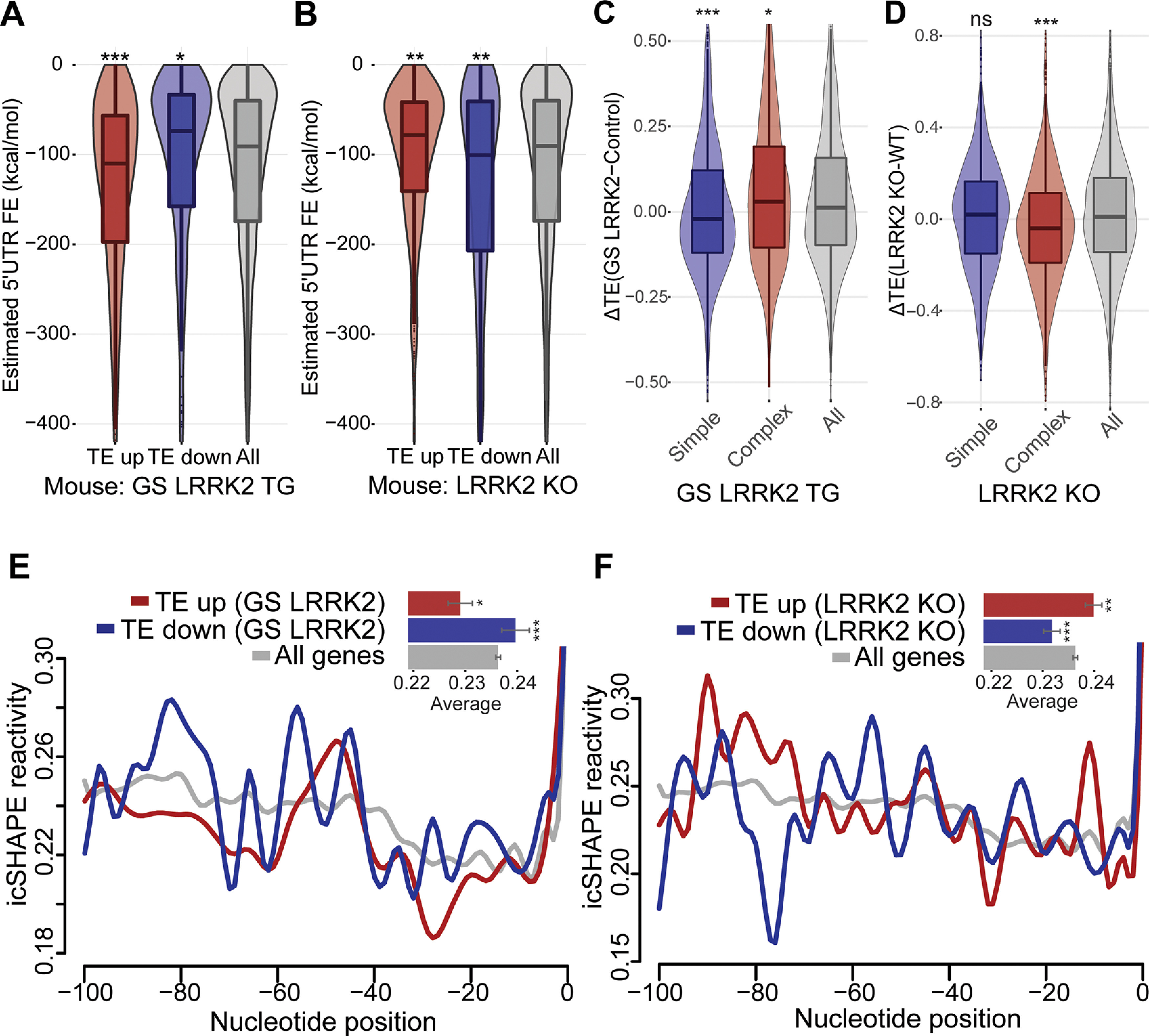
5′UTR secondary structure mediates translational effects of G2019S LRRK2. ***A***, ***B***, Correlation between estimated 5′UTR folding energy and TE changes in (***A***) GS LRRK2 TG or (***B***) LRRK2 KO. Box plot overlaid with violin plot visualizes the median, the first and the third quartile along with the data distribution pattern. 5′UTR folding energy for transcripts was retrieved from UCSC genome database (mm9). The same *z* score ±1.5 cutoff was used. Group sizes: GS TG (TE up: 687, TE down: 335), KO (TE up: 596, TE down: 576). Statistical significance was determined by one-way ANOVA with Bonferroni correction. ***C***, ***D***, Genes with complex 5′UTR secondary structure (estimated folding energy: <–250 kcal/mol, 1145 genes) or simple 5′UTR secondary structure (>–20 kcal/mol, 1036 genes) were selected, and the TE differences between (***C***): GS LRRK2 TG mice and control mice (***D***): LRRK2 KO mice and WT mice were plotted. Statistical significance was tested with Wilcoxon signed-rank test [***C***: *p* < 0.001 (simple), *p* = 0.03533 (complex); ***D***: *p* = 0.6007 (simple), *p* < 0.001 (complex)]. 3′UTR structures do not show correlation (Extended Data Fig. 2-1). ***E***, ***F***, Differential icSHAPE reactivity profiles between TE up and TE down genes. The same TE up and TE down genes with *z* score ±1.5 were used; (***E***) GS TG (TE up: 687, TE down: 335), (***F***) LRRK2 KO (TE up: 596, TE down: 576). icSHAPE data from mouse ES cells were extracted (Spitale et al., 2015), and a window of −100–0 nt 5′ of start codon (CDS start) was used. Average icSHAPE reactivity values: all genes: 0.236, TE up (GS): 0.229, TE down (GS): 0.240, TE up (KO): 0.237, TE down (KO): 0.219. Statistical significance (compared with all genes) was measured by non-parametric Mann–Whitney test. Error bars indicate SEM, *p < 0.05, **p < 0.01, ***p < 0.001.

10.1523/ENEURO.0310-21.2021.f1-2 Extended Data Figure 1-2 Ribosome profiling expression analysis results. Download Figure 1-2, XLS file.

We characterized translation by ribosome profiling, the deep sequencing of ribosome-protected mRNA fragments generated by nuclease digestion. Ribosome profiling provides a quantitative measurement of translation and reports on the precise location of translating ribosomes across the transcriptome (Ingolia et al., 2009). We inferred the translational activity of different mRNAs by calculating the TE, the ratio between the abundance of ribosome footprints derived from a gene to the overall abundance of its mRNA as determined by RNA-seq (Anders and Huber, 2010; Brar and Weissman, 2015; Ingolia, 2016). Comparison of the global distribution of TE values between LRRK2 transgenic mice and non-transgenic littermate control mice revealed broad alterations in TE distribution (Fig. 1*B*). Likewise, LRRK2 KO mice showed widespread differences in TE relative to wild-type control mice (Fig. 1*C*). In contrast, G2019S/D1994A LRRK2 transgenic mice have a TE distribution similar to those in non-transgenic control mice, indicating that the changes in LRRK2 transgenic mice are dependent on kinase activity (Fig. 1*D*; Greggio et al., 2006; Smith et al., 2006). The broadly altered TE distribution indicates that G2019S LRRK2 causes increased expression of some genes (TE up) and decreased expression of others (TE down), distorting the overall translatome.

It has been shown that G2019S LRRK2 enhances the translation of transcripts containing complex 5′UTR structure (Kim et al., 2020). Therefore, we compared the predicted 5′UTR folding energy between genes showing elevated or reduced TE from each comparison. The TE up genes in G2019S LRRK2 transgenic mouse brain have significantly lower folding energy than randomly selected control genes with the same group size (Fig. 2*A*), indicating that they have more complex 5′UTR secondary structures. Conversely, the TE down genes have significantly higher folding energy compared with the control genes, which suggests that they have less structured 5′UTR (Fig. 2*A*). Notably, LRRK2 KO mice show the reverse trend, indicating that loss of LRRK2 has the opposite effect from hyperactive G2019S LRRK2 (Fig. 2*B*). The same trend is clear when we stratify transcripts according to the strength of their 5′UTR secondary structure (Fig. 2*C*,*D*). Unlike the case for 5′UTRs, 3′UTR folding energy does not show LRRK2-dependent correlation with TE (Extended Data Fig. 2-1*A*,*B*). In addition, we did not find significant TE changes from 5′ terminal oligopyrimidine (TOP)-containing genes, which are known to be regulated by phosphorylation of 4E-BP (Extended Data Fig. 2-1*C*,*D*; Thoreen et al., 2012). Therefore, our ribosome profiling data from the mouse brain samples indicate that LRRK2 enhances translation of mRNAs with complex 5′UTR secondary structure in a kinase activity-dependent manner.

10.1523/ENEURO.0310-21.2021.f2-1 Extended Data Figure 2-1 3′UTR secondary structure is not related to translational effects of G2019S LRRK2. ***A***, ***B***, 3′UTR secondary structure folding energy differences between TE up and TE down genes (standard *z* score ±1.5 was used). Unlike the 5′UTR folding energy comparison, 3′UTR folding energy did not show opposing directions of effects between G2019S (GS) LRRK2 transgenic (TG) and LRRK2 KO mice. Statistical significance was determined using Wilcoxon signed-rank test [***A***, *p* = 0.002338 (TE up), *p* = 0.02327 (TE down); ***B***, *p* = 0.01194 (TE up), *p* = 0.0254 (TE down)]. ***C***, ***D***, TE differences of 5′ TOP mRNAs in LRRK2 mouse models. Wilcoxon signed-rank test; ***C***, *p* = 0.2112; ***D***, *p* = 0.09034. background signal. Error bars indicate SEM, *p < 0.05, **p < 0.01, ns = no significance. Download Figure 2-1, TIF file.

Recent advances in molecular techniques that combine chemical probes and deep sequencing have provided transcriptome-wide measurements of RNA structure in living cells. We analyzed mouse RNA structure data (icSHAPE; Spitale et al., 2015) to estimate basal levels of 5′UTR structural complexity of genes differentially regulated by LRRK2. Low icSHAPE signal indicates low chemical reactivity at a given nucleotide, thereby suggesting a higher likelihood that it participates in secondary structures in cells. We compared icSHAPE reactivity between TE up and TE down genes from G2019S LRRK2 transgenic and LRRK2 KO mice. Structure probing data from 100 nt windows 5′ of the CDS start site revealed that the TE up genes in G2019S LRRK2 have significantly low average icSHAPE reactivity (0.229) associated with more complex structure, while the TE down genes have higher average reactivity (0.240) suggesting low structural complexity (Fig. 2*E*). LRRK2 KO mice show the opposite trend (up: 0.237, down: 0.219; Fig. 2*F*). These results suggest that the 5′UTR secondary structure adjacent to the start codon may play a role in the translatome alteration by G2019S LRRK2.

**Figure 3.**
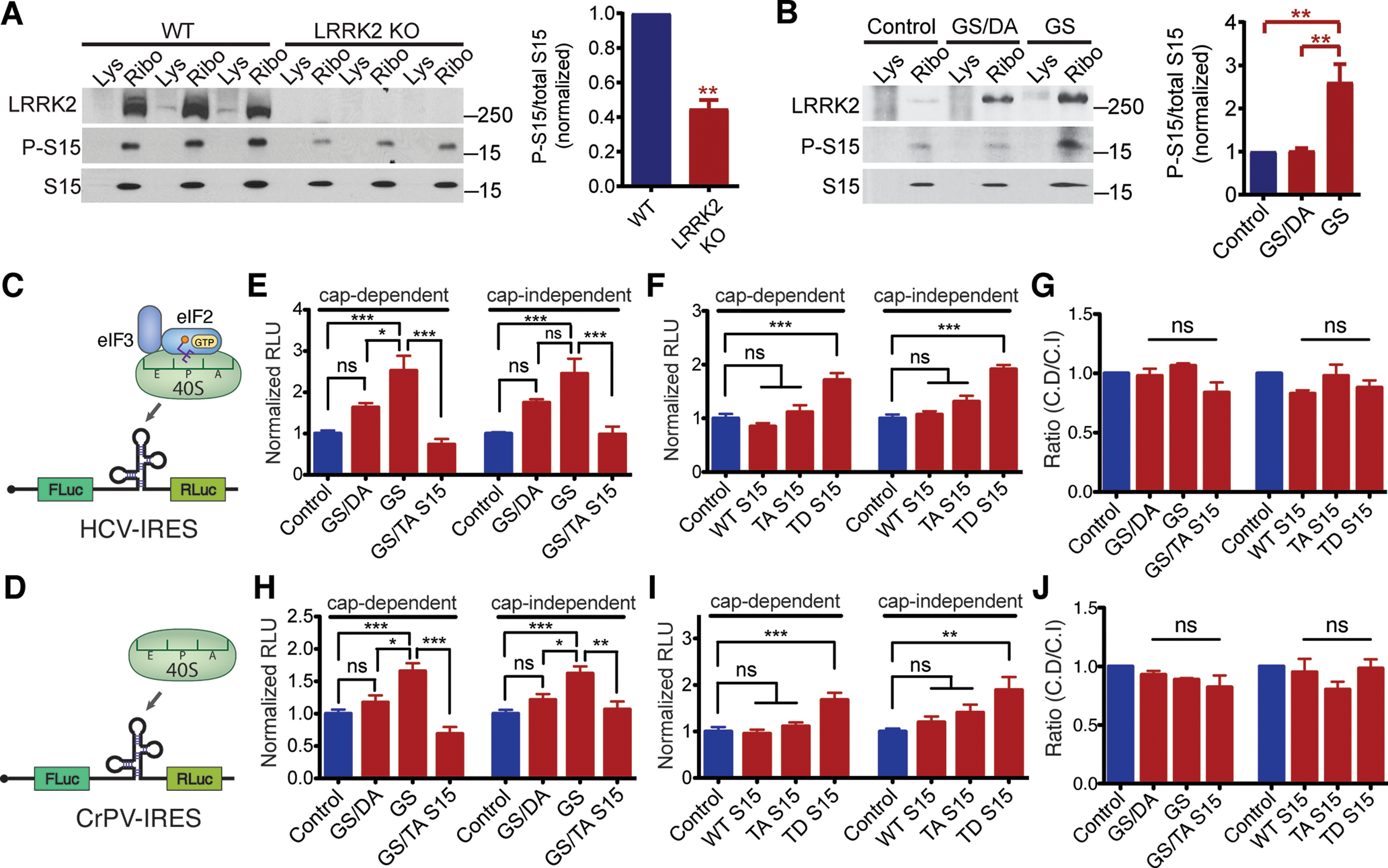
G2019S LRRK2 increases mRNA translation independent of initiation factors. ***A***, ***B***, Western blotting and quantification of T136 S15 phosphorylation in the mouse brain. LRRK2 KO (***A***) and G2019S LRRK2 transgenic (***B***) mice. Whole-brain lysate was used. *n* = 3, biological replicates. Statistical significance was determined by (***A***) unpaired *t* test (***B***) one-way ANOVA with Bonferroni correction. ***C***, ***D***, Schematics of HCV-IRES and CrPV-IRES reporters. ***E–G***, HCV-IRES reporter assays. ***C***, *n* = 4; ***D***, *n* = 3, respectively. ***H–J***, CrPV IRES reporter assays. ***F***, *n* = 4; ***G***, *n* = 3, respectively. Reporter assays were performed in primary mouse cortical neurons with transient transfection, and each experiment is an average of triplicates. All values were divided by the average of control values. Reporter mRNA levels were controlled (Extended Data Fig. 3-1). WT, wild type; Fluc, firefly luciferase; RLuc, *Renilla* luciferase; RLU, relative light units. Statistical significance was determined by one-way ANOVA with Bonferroni correction. Error bars indicate SEM, *p < 0.05, **p < 0.01, ***p < 0.001, ns = no significance.

Translation initiation is a tightly regulated process, with many eukaryotic initiation factors (eIFs) involved in the regulation and facilitation of the process (Sonenberg and Hinnebusch, 2009; Jackson et al., 2010). Of note, DEAD-box RNA helicases including eIF4A, Ddx3, and Dhx29 are thought to resolve 5′UTR secondary structure of mRNAs with the help of other initiation factors such as eIF4B (Parsyan et al., 2011; Sen et al., 2015). Previous studies suggested that T136 phosphorylation of ribosomal protein S15 (uS19) mediates the translational effects of G2019S LRRK2 (Martin et al., 2014b; Kim et al., 2020). Consistent with this, we found that S15 T136 phosphorylation is increased in the G2019S LRRK2 transgenic mouse brain and decreased in the LRRK2 KO mouse brain (Fig. 3*A*,*B*). To investigate potential crosstalk between G2019S LRRK2, phosphorylated S15 and eIFs, we employed bicistronic reporters with hepatitis C virus (HCV) or cricket paralysis virus (CrPV) IRES. HCV-IRES and CrPV-IRES do not require RNA helicase activity to initiate translation, and CrPV-IRES initiation is entirely independent of eIFs (Fig. 3*C*,*D*; Jackson et al., 2010). In these bicistronic reporter assays, cap-dependent translation of firefly luciferase is dependent on helicase activity of eIFs, while IRES-driven cap-independent translation of *Renilla* luciferase is helicase independent. Unexpectedly, both IRES reporters show the same cap-dependent and cap-independent translational induction by G2019S LRRK2 and T136D S15, thereby leaving the ratios between cap-dependent and cap-independent translation unchanged (Fig. 3*E–J*; Extended Data Fig. 3-1). Since the CrPV IRES does not require any initiation factors to recruit ribosomes, the results indicate that the translational effects of G2019S LRRK2 are independent of translation initiation factors, and phosphorylation of S15 is sufficient to enhance the translation of mRNAs with structured 5′UTRs.

10.1523/ENEURO.0310-21.2021.f3-1 Extended Data Figure 3-1 Reporter transcript levels for IRES reporter assays. ***A***, ***B***, qPCR measurement of luciferase transcript levels in IRES reporter assays. One-way ANOVA with Bonferroni correction was used, and there were no significant changes in the reporter transcript levels detected; **p < 0.01, ***p < 0.001, ns = no significance. Download Figure 3-1, TIF file.

**Figure 4.**
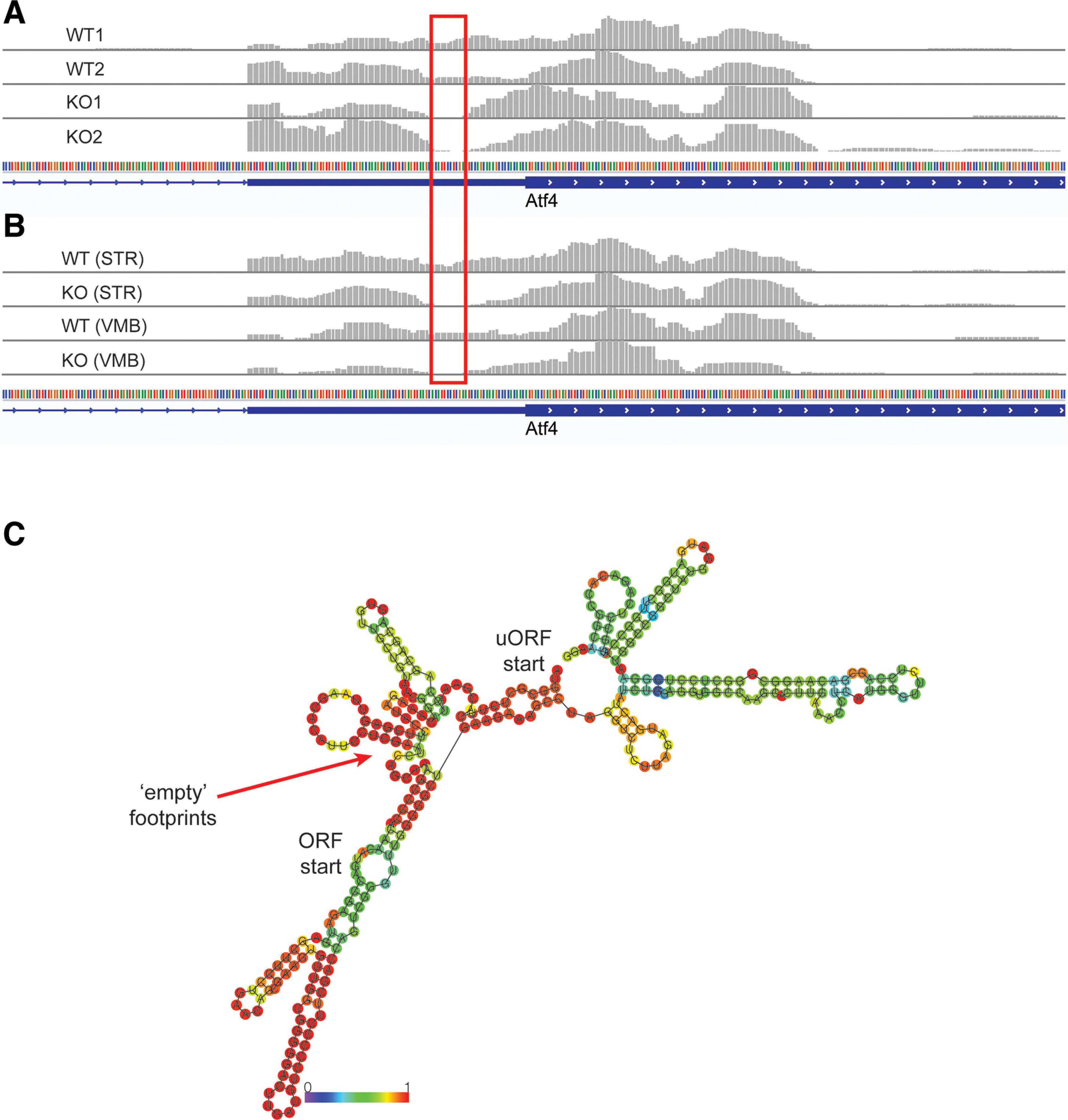
Ribosome footprint distributions on Atf4 uORFs in the LRRK2 KO brain. ***A***, ***B***, Ribosome footprints distribution in the 5′UTR of *Atf4* gene (visualized: chr15:80,086,569–80,086,862). Red box indicates the region that ribosomes are depleted in the LRRK2 KO brain. ***C***, RNA structure prediction of the *Atf4* uORF sequences by ViennaRNA RNAfold (Lorenz et al., 2011). The regions of depleted ribosome footprints have high probability to form secondary structure. In addition, relationship between G2019S LRRK2 and eIF2ɑ was addressed (Extended Data Fig. 4-1).

While we sought to characterize translational abnormalities in the LRRK2 KO mouse brain, we found unexpected patterns of ribosomal footprint distribution on the *Atf4* upstream open reading frame (ORF) regions. *Atf4* is the key transcription factor underlying one branch of the integrated stress response (ISR) pathway and its expression is known to be translationally regulated (Vattem and Wek, 2004). eIF2ɑ-mediated regulation of *Atf4* is a well-studied example of translational regulation using termination-reinitiation balance between the upstream ORFs. We observed that in the LRRK2 KO brain, ribosome footprints are depleted 15–20 nt before the start codon of the main ORF (Fig. 4*A*). We performed additional ribosome profiling experiments with the caudate putamen (striatum; STR) and the ventral midbrain (VMB) of LRRK2 KO mouse brain and found that the footprint depletion is consistent across all ribosome profiling experiments conducted (Fig. 4*B*). Since we found that 5′UTR secondary structure is important to LRRK2-mediated translational regulation, we examined potential secondary structures near the depleted region. Computational secondary structure predictions (RNAfold) reported multiple potential hairpins in the *Atf4* mRNA, and the depleted region in particular has a very high probability to form hairpin (Fig. 4*C*). These results further point to the importance of 5′UTR secondary structure near start codon for the translational effects of LRRK2.

10.1523/ENEURO.0310-21.2021.f4-1 Extended Data Figure 4-1 Delayed ISR recovery from G2019S LRRK2-expressing neurons. ***A***, Phosphorylation of eIF2ɑ in the G2019S LRRK2 transgenic brains. Dissected striatal tissues, age three to four months, *n* = 3, biological replicates. ***B***, Mouse cortical neurons were prepared from pregnant transgenic breeders at E15. Pups were separated and individually genotyped. Control: wild type or single transgenic (CaMKII-tTA or tet-G2019S LRRK2), G2019S LRRK2: double transgenic. Tg: thapsigargin (1 μm). *, background signal. Download Figure 4-1, TIF file.

**Figure 5.**
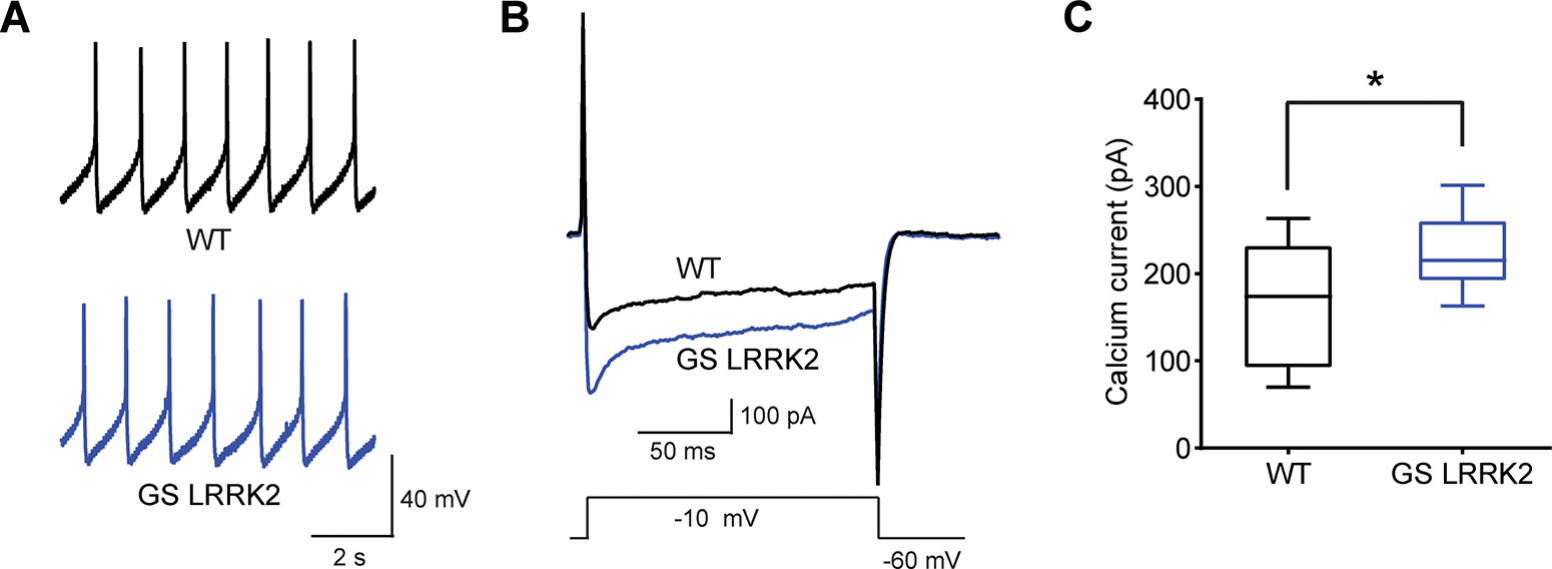
Calcium currents recorded in SNpc DA neurons. ***A***, Comparison of spontaneous AP firing pattern of DA neurons between wild-type and GS LRRK2 mouse slices. ***B***, Calcium currents were measured in mouse SNpc DA neurons using whole-cell patch clamp recordings. ***C***, Quantification of calcium peak currents. Data are expressed as means ± SEM, *n* = 12 slices from 12 animals for each group. Intrinsic properties were measured (Extended Data Fig. 5-1).

Since *Atf4* uORF footprint abnormality is observed in the LRRK2 KO brain and *Atf4* induction is a central downstream pathway of ISR, we further sought to determine a potential relationship between G2019S LRRK2 and ISR. First, eIF2ɑ phosphorylation levels in the G2019S LRRK2 transgenic brain were examined. We found no steady-state induction of eIF2ɑ phosphorylation regardless of the transgene expression levels (Extended Data Fig. 4-1*A*). Next, we tested the potential relationship by inducing ISR in G2019S LRRK2-expressing primary neurons cultured from the transgenic mouse model. Of note, G2019S LRRK2-expressing neurons have defective recovery from thapsigargin-induced ISR (Extended Data Fig. 4-1*B*). Considering the enhanced translation of structured 5′UTR-containing transcripts in the G2019S LRRK2 brain, this defected recovery could be because of translational defects caused by G2019S LRRK2 inhibiting 5′UTR-mediated translational regulation required for ISR recovery. In addition, since thapsigargin induces ISR by blocking SERCA, defective calcium handling in G2019S LRRK2 neurons could also exacerbate ISR. A previous study suggested that dysregulated translation leads to increased calcium influx in G2019S LRRK2 human dopamine neurons (Kim et al., 2020). In this regard, we performed calcium recordings with the G2019S LRRK2-expressing brain. Basic electrophysiological properties including spontaneous and evoked AP wave of SNpc dopamine neurons are indifferent to G2019S LRRK2 expression in the brain (Extended Data Fig. 5-1). Substantia nigra dopamine neurons also show similar pacemaking activities compared with the wild type (Fig. 5*A*). However, calcium currents measurement showed significant increase of calcium currents in the G2019S LRRK2-expressing brain (Fig. 5*B*,*C*). These results are consistent with the previous report of increased calcium influx in G2019S LRRK2 human dopamine neurons (Kim et al., 2020).

10.1523/ENEURO.0310-21.2021.f5-1 Extended Data Figure 5-1 Intrinsic properties of mouse brain DA neurons. ***A***, Summary of electrophysiological characteristics of DA neurons in SNpc during recordings, including pipette resistance (Rp), input resistance (Rin), series resistance (Rseries), leak currents (Leak), and resting membrane potential. ***B***, Spontaneous AP firing pattern in DA neurons. ***C***, A representative single AP wave with a half width of 2 ms. ***D***, Evoked APs. The presence of a sag (arrow) in the membrane potential and APs were detected in current-clamp immediately after rupturing the membrane. ***E***, Immunofluorescence image showing recorded neurons are TH-positive. Alexa Fluor 568 was injected to label recorded neurons. Scale bar: 50 μm. Data are expressed as means ± SEM, WT, *n* = 6 slices from 6 mice; GS LRRK2, *n* = 6 slices from 6 mice. Download Figure 5-1, TIF file.

## Discussion

In this study, we found that G2019S LRRK2 alters the global translational landscape in the mouse brain. Dysregulated translation caused by G2019S LRRK2 has been reported in *Drosophila* models and human dopamine neurons differentiated from patient-derived iPSCs (Martin et al., 2014b; Kim et al., 2020). Our data from the mouse brain are in line with the previous results showing that G2019S LRRK2 induces genome-wide translational abnormality. In addition, the 5′UTR-mediated translational shift, which was previously observed in the G2019S LRRK2 human dopamine neurons, is also present in the mouse brain. In G2019S LRRK2 transgenic mice, mRNAs with complex 5′UTR secondary structure tend to have elevated TE, while LRRK2 KO mice show the opposite trend. Analysis of RNA secondary structure data suggest that mRNA secondary structure on the 5′UTR regions near the start codon is important for these TE changes. These observations are in accordance with the previous finding that G2019S LRRK2 alters genome-wide translation by favoring mRNAs with complex 5′UTR secondary structure.

While the precise structural mechanism underlying the 5′UTR-mediated mRNA preference is unclear, our IRES reporter assays suggest that the enhanced translation in G2019S LRRK2 expressing neurons does not rely on translation initiation factors. Considering that G2019S LRRK2 is known to phosphorylate multiple ribosomal proteins including S15, our results bolster the idea that phosphorylation of ribosomal proteins could change the global translational landscape autonomously. Of note, the IRES reporter assays also indicate that the effects of LRRK2 may not be limited to translational initiation, since IRES-recruited ribosomes are thought to bypass scanning (Jackson et al., 2010). While our analyses indicate a strong correlation between 5′UTR secondary structure and TE, an alteration in secondary structure may in theory impact elongation as well. Secondary structure-mediated regulation is generally considered in the context of translation initiation since the coding region has limited degree of freedom for nucleotide-based secondary structure formation. However, as the *Atf4* CDS secondary structure prediction depicts, it is possible that elongation could be, at least partially, regulated by mRNA secondary structure as well. Therefore, these collectively suggest that the G2019S LRRK2 mutation and its downstream effects can facilitate translation during both initiation and elongation steps if secondary structure-mediated regulation is in place.

Ribosome footprint depletion at the *Atf4* 5′UTR in LRRK2 KO provides new information on the mechanisms by which LRRK2 affects translation. It suggests that the low TE of complex 5′UTR genes in the LRRK2 KO brain is because of strong hairpin formation and reduced ribosomal processivity. Alternatively, it is possible that the depletion is caused by disome formation, which can reduce the recovery of footprints in ribosome profiling experiments that include a monosome-specific size-selection step. This disome hypothesis is supported by the facts that the depletion is just in front of the main CDS start codon, and the main CDS also tends to form strong hairpin structure right after the start codon (Fig. 4*C*). Both cases are consistent with the interpretation of reduced ribosomal processivity in the LRRK2 KO brain. It further suggests that uORF-mediated regulation of *Atf4* expression could potentially be regulated by manipulating ribosomal processivity. While we did not find any *Atf4* footprint distribution abnormality in the G2019S LRRK2 transgenic brain, we cannot exclude the possibility that increased ribosomal processivity could impair the ISR, thereby incurring a long-term cellular stress in G2019S LRRK2 PD. Delayed recovery from thapsigargin-mediated ISR in G2019S LRRK2 neurons might be linked to this increased processivity. Since ATF4 plays central roles in the integrated stress responses, including induction of genes necessary to cope with cellular stresses, understanding the exact molecular mechanisms for *Atf4* expression regulation will deepen our knowledge on the pathobiology of LRRK2 PD.

Since this study was conducted with dissected brain tissues without cell-type specificity, dopamine neuron-specific translational profiling experiments in rodent models, which have been done in human iPSC-derived dopamine neuron and *Drosophila* models, would further reveal the specific changes relevant to G2019S LRRK2 PD (Kim et al., 2020; Pallos et al., 2021). In addition, there is a recent report suggesting that G2019S LRRK2 leads to reduced bulk translation in rodent neurons (Deshpande et al., 2020). The study was conducted with different model systems from this study (cultured neurons, *in vitro* translation system, and skin fibroblasts), which makes it hard to directly compare the results. However, bulk protein synthesis rate is tightly related to the neuronal activity levels. In this regard, investigating the relationship between LRRK2 and neuronal activity would be informative to collectively comprehend the molecular mechanisms of LRRK2-mediated translational regulation.

It is noteworthy that calcium influx is increased in the substantia nigra dopamine neurons in the G2019S LRRK2-expressing brain. The increased calcium influx was originally reported in G2019S LRRK2 human dopamine neurons. While the previous findings from cultured neurons initiated a plausible molecular mechanism that can led to a long-term dopamine neuronal stress, the electrophysiological characteristic of a neuron is heavily influenced by the neuron’s wiring context. Therefore, monitoring calcium dynamics in a fully developed adult brain tissue is essential to validate the hypothesis (Yin et al., 2021). In this manner, our findings on the increased calcium influx *in vivo* bolster the suggested molecular etiology that calcium dysregulation leads to dopamine neuronal stress in the G2019S LRRK2 PD.

## Synthesis

Reviewing Editor: Laura Volpicelli-Daley, University of Alabama at Birmingham Decisions are customarily a result of the Reviewing Editor and the peer reviewers coming together and discussing their recommendations until a consensus is reached. When revisions are invited, a fact-based synthesis statement explaining their decision and outlining what is needed to prepare a revision will be listed below. The following reviewer(s) agreed to reveal their identity: Yi-Shuian Huang, Jeremy Nichols.

Both reviewers felt the study adds additional understanding of post-transcriptional regulation in the context of G2019S-LRRK2, kinase dead LRRK2 and LRRK2 KO.The study expands on previous findings from differentiated iPSCs into mouse putamen which has more physiological relevance. Overall, the study provides interesting findings on the role of G2019S-LRRK2 in phosphorylation in RPS15, translation of mRNAs with complex 5’UTR and calcium signaling in the nigra.

Both reviewers had specific comments regarding further characterizaton of the mouse models. The reviewers also asked for clarification and more details in the methods sections. In addition, how does the impact of LRRK2 compare to known modulators of disruptors of translation.

Importantly, the RNA sequencing results should be deposited in the public domain before publication.

Specifically:

1) Please provide provide further validation of the models by immunoblot comparing expression levels of LRRK2 on a single gel as well as immunoblot validation of additional markers of LRRK2 kinase activity such as pRab substrates.

2) Is this degree of change in translational efficiency in G2019S mice similar to other cellular responses to stress?

For method clarification:

1) Please provide more detailed descriptions of the mice: clearly describe the source of mice, how long they removed doxycycline-containing diet or water to induce transgene expression and how high the transgene is expressed in comparison to control (Is the control transgenic Camk2-tTA?).

2)The luciferase assays are described ambiguously. Were Luc reporter plasmids transfected along with plasmids expressing LRRK2 GS/DA, GS alone, GS/TA with S15 into CD1 wild-type cortical neurons? What were the LRRK2 plasmids, the S15 expression plasmids and what were the ratios of each used? What is the control plasmid? It is set to 1, how? How were the data normalized?

3) Within these ribosome profiling data, are there methods available to determine if there are differences in processivity across all transcripts?

The reviewers also requested more thorough analyses/interpretations which can be addressed in the discussion section and modifications for the figure legends. These can be found bleow in the specific comments.

Reviewer 1:

In previous studies from Dawson lab, LRRK2 phosphorylates RPS15 and promotes translation of mRNAs with complex 5’-UTR. In dopaminergic neurons differentiated from G2019S LRRK2-hiPSCs, mRNAs with complex secondary structure in the 5’-UTR are translated more efficiently, so some increased proteins induced by G2019S LRRK2 enhance calcium influx and contribute to the pathogenesis of Parkinson’s disease. In this manuscript, the authors have extensively investigated ribosome footprinting profiles by using caudate putamen tissues from 3-4-mo-old LRRK2-KO, G2019S LRRK2, G2019SD1994A (kinase-dead mutant), wild-type and transgenic control mice. Their findings in translatomes and elevated calcium currents in SNpc DA neurons of G2019S LRRK2 mice are in the agreement with the literature findings. Moreover, the reporter assays also support that phosphorylation of T136 in RPS15 by LRRK2 accounts for enhanced cap-dependent and -independent translation. Overall, this is a nice study to comprehensively characterize how gain-of-function and loss-of-function LRRK2 affects translational landscapes in mouse brain tissues. There are several points needed to be clarified or discussed before I can recommend it for publication.

Specific comments:

1. Although the authors cited Xiong et al 2017 and Nikonova 2012 studies and likely obtained these mice from both labs or Jackson Laboratory, they should clearly describe the source of mice, how long they removed doxycycline-containing diet or water to induce transgene expression and how high the transgene is expressed in comparison to control (Is the control transgenic Camk2-tTA?). Please describe sufficiently about mouse information.

2. Figure 1C and 1D appear to be mislabeled. Please switch 1C to 1D and 1D to 1C. For Fig. 1-1, the triple periodicity in some samples, such as control 3, TG_2, WT and KO,

is not as good as the others. Any reasons? Would the unequal triple periodicity among samples affect the results? Some discussions may help readers to understand how to judge ribosome footprinting data.

3. In Figure 4, the authors found that LRRK2-KO caused ribosome depletion in the 15-20 nucleotides upstream of the start codon of the main Atf4 ORF. RNAfold predicted a complex structure in this region. This is a very interesting finding despite of lacking mechanisms. I don’t understand why the formation of disomes on a complex RNA region cannot be separated by RNase digestion. Can the authors explain more? Are the 15-20 nucleotides located in the last uORF of Atf4? Moreover, how common is this phenomenon (ribosome-depleted) in transcripts with a complex 5’-UTR structure or with many uORFs? Please also list others genes with such a pattern in their 5’-UTR.

4. The RNA sequencing results should be deposited in the public domain before publication.

Reviewer 2

This manuscript seems to add to a recent publication from iPSCs describing similar effects on translation by mutant LRRK2 (of which, the specific overlap or discrepancies between the two species could be fleshed out more here). I am not sure there is a direct link between S15 phosphorylation-translational changes-Ca++ signaling, though this is not directly stated. The manuscript adds additional understanding of post-transcriptional regulation in the context of LRRK2 mutant and KO, and then provides experiments to support a role of ribosomal protein S15 and an effect in nigral calcium signaling as a potential cause. There are several questions and some minor points that should be addressed prior to publication.

•The effect of LRRK2 modulation on translational efficiency is described as broad. What is the effect size of LRRK2 related changes in translational efficiency compared to a known disruptor of translation? Is this degree of change in translational efficiency similar to other cellular responses to stress, i.e. is this a G2019S induced stress environment where broad alterations in translational efficiency of complex RNAs is shifted to stress related genes?

• The only characterization of the animals used in the study are the immunoblots in Figure 3, could the authors provide further validation of these models by immunoblot comparing expression levels of LRRK2 and modification of substrates on a single gel? The authors should provide immunoblot validation of additional markers of LRRK2 kinase activity such as pRab substrates alongside the validation of pS15 substrate.

•The luciferase assays are described ambiguously. Were Luc reporter plasmids transfected along with plasmids expressing LRRK2 GS/DA, GS alone, GS/TA with S15 into CD1 wild-type cortical neurons? What were the LRRK2 plasmids, the S15 expression plasmids and what were the ratios of each used? What is the control plasmid? It is set to 1, how? How were the data normalized? The authors should test for significant differences in Luc activity between GS and GS/DA. If the authors were to immunoblot the neuron cultures, or a surrogate cell system, would the phosphorylation of S15 track with LRRK2 kinase activity? If this data holds then does this reduce the confidence in LRRK2 regulation of translation via phosphorylation of 4EBP?

•Within these ribosome profiling data, are there methods available to determine if there are differences in processivity across all transcripts?

•If translational efficiency is increased in LRRK2 G2019S and it is mediated through hyper phosphorylation of S15 (thus dysregulating its function), and LRRK2 KO leads to a reduced translational efficiency, does this indicate a typical/normal, non-mutant regulatory role for LRRK2 phosphorylation of S15 such that in the absence of S15 phosphorylation, translational efficiency decreases? Would S15 Thr136Ala knockin resolve the differences in translational efficiency imparted by G2019S LRRK2? The authors should also cite Deshpande et al FASEB and Pallos et al NBD and integrate their findings in the discussion.

## Author Response

Synthesis of Reviews:

Synthesis Statement for Author (Required):

Both reviewers felt the study adds additional understanding of post-transcriptional regulation in the context of G2019S-LRRK2, kinase dead LRRK2 and LRRK2 KO. The study expands on previous findings from differentiated iPSCs into mouse putamen which has more physiological relevance. Overall, the study provides interesting findings on the role of G2019S-LRRK2 in phosphorylation in RPS15, translation of mRNAs with complex 5’UTR and calcium signaling in the nigra.

Both reviewers had specific comments regarding further characterization of the mouse models. The reviewers also asked for clarification and more details in the methods sections. In addition, how does the impact of LRRK2 compare to known modulators of disruptors of translation.

The LRRK2 transgenic mice were fully characterized in PMID 29386392 and thus we did not repeat or further characterize the mice here. We have expanded the methods section to include additional details of the mouse models as suggested. The translational phenotypes induced by G2019S LRRK2 are different from conventional translational responses including integrated stress response (ISR); and do not share signaling cascades (phosphorylation of eIF2ɑ).

Importantly, the RNA sequencing results should be deposited in the public domain before publication.

The RNA sequencing results were deposited and the accession number is GSE167704. This information may not have been available because then the blind for the reviewers would have been broken.

Specifically:

1) Please provide provide further validation of the models by immunoblot comparing expression levels of LRRK2 on a single gel as well as immunoblot validation of additional markers of LRRK2 kinase activity such as pRab substrates.

Here we provide our published data presented in (Xiong et al., 2017). Previously, we published an immunoblot showing LRRK2 expression levels and autophosphorylation levels of LRRK2 in the transgenic mouse brain. We argue that LRRK2 autophosphorylation is a less biased way to show the kinase activity of LRRK2 than testing any reported kinase substrates, since autophosphorylation does not depend on potential complications for the enzyme-substrate reaction in vivo (e.g. expression levels, co-factors, and localization).

(From Figure 1) (Xiong et al., 2017)

2) Is this degree of change in translational efficiency in G2019S mice similar to other cellular responses to stress?

Regarding the magnitude of translational changes, integrated stress response (ISR) triggers robust global translational shutdown via phosphorylation of eIF2ɑ and it is generally shown by collapse of polysomes. Our group previously showed that G2019S LRRK2 expression in the Drosophila brain leads to ∼25% of bulk protein synthesis increase (Martin et al., 2014). Although this is a noticeable change to the total protein synthesis, we do not think that the magnitude of G2019S LRRK2-mediated changes is as dramatic as the ISR’s. As discussed in the manuscript, our interpretation is rather a mild distortion yet affecting many genes with strong 5'UTR secondary structures.

Regarding a potential mechanistic crosstalk between the ISR and G2019S LRRK2, we checked eIF2ɑ phosphorylation levels in the G2019S LRRK2 transgenic brain with different transgene expression levels and found no steady-state induction of phosphorylation.

(Figure 4-1A, revised manuscript)

While the basal levels of P- eIF2ɑ are unchanged, we further explored the potential relationship by inducing ISR in G2019S LRRK2-expressing primary neurons, which were cultured from the transgenic mouse model. We found that G2019S LRRK2-expressing neurons have defective recovery from thapsigargin-induced ISR. This could be due to 1) defective calcium handling in G2019S LRRK2 neurons as previously reported (Kim et al., 2020) 2) translational defects caused by G2019S LRRK2 inhibiting 5'UTR-mediated translational regulation required for ISR recovery. These results are updated and discussed in the revised manuscript.

(Figure 4-1B, revised manuscript)

For method clarification:

1) Please provide more detailed descriptions of the mice: clearly describe the source of mice, how long they removed doxycycline-containing diet or water to induce transgene expression and how high the transgene is expressed in comparison to control (Is the control transgenic Camk2-tTA?).

Detailed procedures have been updated to the manuscript. Doxycycline-containing diet was removed after weaning. Transgene expression levels were originally characterized as 10-15x compared to the non-transgenic (Xiong et al., 2017). Control transgenic mice were single transgenic (CaMKII-tTA or tet-LRRK2 only) mice.

2)The luciferase assays are described ambiguously. Were Luc reporter plasmids transfected along with plasmids expressing LRRK2 GS/DA, GS alone, GS/TA with S15 into CD1 wild-type cortical neurons? What were the LRRK2 plasmids, the S15 expression plasmids and what were the ratios of each used? What is the control plasmid? It is set to 1, how? How were the data normalized?

Detailed methods and procedures are now in included in the manuscript. CD1 wild-type neurons were transfected. Luciferase reporter to LRRK2/S15 expression plasmid ratio was 1:3. Empty expression plasmids (the vector used for LRRK2 or S15 transgene) were used for control transfection. The values are ratio to the average value of the control (all values in the experiment were divided by the average of the control values), which does not affect statistical analysis.

3) Within these ribosome profiling data, are there methods available to determine if there are differences in processivity across all transcripts?

Unfortunately, there is no available method to determine ribosomal processivity from existing ribosome profiling datasets. Ribosome profiling reports the locations of ribosomes along transcripts. To assess processivity, elongation of ribosomes needs to be measured. This requires an additional way to track ribosomes on the same transcript along multiple timepoints.

The reviewers also requested more thorough analyses/interpretations which can be addressed in the discussion section and modifications for the figure legends. These can be found below in the specific comments.

Reviewer 1:

In previous studies from Dawson lab, LRRK2 phosphorylates RPS15 and promotes translation of mRNAs with complex 5'-UTR. In dopaminergic neurons differentiated from G2019S LRRK2-hiPSCs, mRNAs with complex secondary structure in the 5'-UTR are translated more efficiently, so some increased proteins induced by G2019S LRRK2 enhance calcium influx and contribute to the pathogenesis of Parkinson's disease. In this manuscript, the authors have extensively investigated ribosome footprinting profiles by using caudate putamen tissues from 3-4-mo-old LRRK2-KO, G2019S LRRK2, G2019SD1994A (kinase-dead mutant), wild-type and transgenic control mice. Their findings in translatomes and elevated calcium currents in SNpc DA neurons of G2019S LRRK2 mice are in the agreement with the literature findings. Moreover, the reporter assays also support that phosphorylation of T136 in RPS15 by LRRK2 accounts for enhanced cap-dependent and -independent translation. Overall, this is a nice study to comprehensively characterize how gain-of-function and loss-of-function LRRK2 affects translational landscapes in mouse brain tissues. There are several points needed to be clarified or discussed before I can recommend it for publication.

We appreciate the reviewer's insightful comments which were very helpful to revise our manuscript.

Specific comments:

1. Although the authors cited Xiong et al 2017 and Nikonova 2012 studies and likely obtained these mice from both labs or Jackson Laboratory, they should clearly describe the source of mice, how long they removed doxycycline-containing diet or water to induce transgene expression and how high the transgene is expressed in comparison to control (Is the control transgenic Camk2-tTA?). Please describe sufficiently about mouse information.

(addressed above)

2. Figure 1C and 1D appear to be mislabeled. Please switch 1C to 1D and 1D to 1C. For Fig. 1-1, the triple periodicity in some samples, such as control 3, TG_2, WT and KO,

is not as good as the others. Any reasons? Would the unequal triple periodicity among samples affect the results? Some discussions may help readers to understand how to judge ribosome footprinting data.

Figures 1C and 1D were corrected. While some results such as control 3 do look like they have different phasing of ribosome footprints, we do not think that these samples actually have 'worse' triplet periodicity. Technical factors such as overall footprint length distribution can affect A site estimation of the footprint, thereby changing distribution of the peaks. Since weak periodicity is generally observed as no triplet-repeating patterns at all, we deem that the periodicity seems to be normal if there is clear repetition of 3-nucleotide spacing regardless of the relative peak height on each position.

3. In Figure 4, the authors found that LRRK2-KO caused ribosome depletion in the 15-20 nucleotides upstream of the start codon of the main Atf4 ORF. RNAfold predicted a complex structure in this region. This is a very interesting finding despite of lacking mechanisms. I don't understand why the formation of disomes on a complex RNA region cannot be separated by RNase digestion. Can the authors explain more? Are the 15-20 nucleotides located in the last uORF of Atf4? Moreover, how common is this phenomenon (ribosome-depleted) in transcripts with a complex 5'-UTR structure or with many uORFs? Please also list others genes with such a pattern in their 5'-UTR.

Standard ribosome profiling procedure includes a size selection step to selectively enrich ribosome footprints from monosomes (26-34 nt in this study, it depends on the nuclease used). All libraries in this study were prepared with monosome selection and therefore cannot be used to investigate disome formation. So it is possible that prominent disome formation causes 'depletion' looking footprinting distribution. The depleted region is in the 2nd (last) uORF. While we agree that genome-wide survey of ribosome-depleted regions in the LRRK2 knockout brain might be informative, considering the scope of this manuscript which focuses on the effects of G2019S LRRK2 on translation in vivo, we think that such an approach would be more appropriate as an independent follow-up study.

4. The RNA sequencing results should be deposited in the public domain before publication.

It was deposited to the NCBI GEO: GSE167704

Reviewer 2

This manuscript seems to add to a recent publication from iPSCs describing similar effects on translation by mutant LRRK2 (of which, the specific overlap or discrepancies between the two species could be fleshed out more here). I am not sure there is a direct link between S15 phosphorylation-translational changes-Ca++ signaling, though this is not directly stated. The manuscript adds additional understanding of post-transcriptional regulation in the context of LRRK2 mutant and KO, and then provides experiments to support a role of ribosomal protein S15 and an effect in nigral calcium signaling as a potential cause. There are several questions and some minor points that should be addressed prior to publication.

•The effect of LRRK2 modulation on translational efficiency is described as broad. What is the effect size of LRRK2 related changes in translational efficiency compared to a known disruptor of translation? Is this degree of change in translational efficiency similar to other cellular responses to stress, i.e. is this a G2019S induced stress environment where broad alterations in translational efficiency of complex RNAs is shifted to stress related genes?

(discussed above)

• The only characterization of the animals used in the study are the immunoblots in Figure 3, could the authors provide further validation of these models by immunoblot comparing expression levels of LRRK2 and modification of substrates on a single gel? The authors should provide immunoblot validation of additional markers of LRRK2 kinase activity such as pRab substrates alongside the validation of pS15 substrate.

(addressed above)

•The luciferase assays are described ambiguously. Were Luc reporter plasmids transfected along with plasmids expressing LRRK2 GS/DA, GS alone, GS/TA with S15 into CD1 wild-type cortical neurons? What were the LRRK2 plasmids, the S15 expression plasmids and what were the ratios of each used? What is the control plasmid? It is set to 1, how? How were the data normalized? The authors should test for significant differences in Luc activity between GS and GS/DA. If the authors were to immunoblot the neuron cultures, or a surrogate cell system, would the phosphorylation of S15 track with LRRK2 kinase activity? If this data holds then does this reduce the confidence in LRRK2 regulation of translation via phosphorylation of 4EBP?

(reporter assay questions were addressed above)

GS/DA and GS comparison statistics were updated on the figures (Figures 3E and 3H).

While the phosphorylation of 4E-BP was originally found in Drosophila models, increased phosphorylation of 4E-BP by G2019S LRRK2 in the mouse brain was not detected (Trancikova et al., 2012). Furthermore, we did not find translational efficiency changes from 5'terminal oligopyrimidine-containing genes, which are regulated by P-4E-BP.

(Figures 2-1, C and D, revised manuscript)

•Within these ribosome profiling data, are there methods available to determine if there are differences in processivity across all transcripts?

(answered above)

•If translational efficiency is increased in LRRK2 G2019S and it is mediated through hyper phosphorylation of S15 (thus dysregulating its function), and LRRK2 KO leads to a reduced translational efficiency, does this indicate a typical/normal, non-mutant regulatory role for LRRK2 phosphorylation of S15 such that in the absence of S15 phosphorylation, translational efficiency decreases? Would S15 Thr136Ala knockin resolve the differences in translational efficiency imparted by G2019S LRRK2? The authors should also cite Deshpande et al FASEB and Pallos et al NBD and integrate their findings in the discussion.

We expect that expression of phosphorylation-deficient mutant of S15 (T136A) in the mouse brain would rescue the 5'UTR-mediated translational effects by G2019S LRRK2, as previously shown in human dopamine neurons (Kim et al., 2020). However, our attempts to generate S15 knock-in mice were not successful and likely the embryos were not viable. The role of T136 S16 phosphorylation during development and in the function of other organs is unknown.

The suggested papers are cited and discussed.

References:

Kim JW, Yin X, Jhaldiyal A, Khan MR, Martin I, Xie Z, Perez-Rosello T, Kumar M, Abalde-Atristain L, Xu J, Chen L, Eacker SM, Surmeier DJ, Ingolia NT, Dawson TM, Dawson VL (2020) Defects in mRNA Translation in LRRK2-Mutant hiPSC-Derived Dopaminergic Neurons Lead to Dysregulated Calcium Homeostasis. Cell Stem Cell 27:633-645.e637.

Martin I, Kim JW, Lee BD, Kang HC, Xu JC, Jia H, Stankowski J, Kim MS, Zhong J, Kumar M, Andrabi SA, Xiong Y, Dickson DW, Wszolek ZK, Pandey A, Dawson TM, Dawson VL (2014) Ribosomal protein s15 phosphorylation mediates LRRK2 neurodegeneration in Parkinson's disease. Cell 157:472-485.

Trancikova A, Mamais A, Webber PJ, Stafa K, Tsika E, Glauser L, West AB, Bandopadhyay R, Moore DJ (2012) Phosphorylation of 4E-BP1 in the mammalian brain is not altered by LRRK2 expression or pathogenic mutations. PLoS One 7:e47784.

Xiong Y, Neifert S, Karuppagounder SS, Stankowski JN, Lee BD, Grima JC, Chen G, Ko HS, Lee Y, Swing D, Tessarollo L, Dawson TM, Dawson VL (2017) Overexpression of Parkinson's Disease-Associated Mutation LRRK2 G2019S in Mouse Forebrain Induces Behavioral Deficits and alpha-Synuclein Pathology. eNeuro 4.
