# Supplementary figures and images for: Dysregulated mRNA Translation in the G2019S LRRK2 and LRRK2 Knock-Out Mouse Brains
Source: eNeuro. 2021 Nov 30;8(6):ENEURO.0310-21.2021. doi: 10.1523/ENEURO.0310-21.2021 (PMC8638676; doi:10.1523/ENEURO.0310-21.2021)

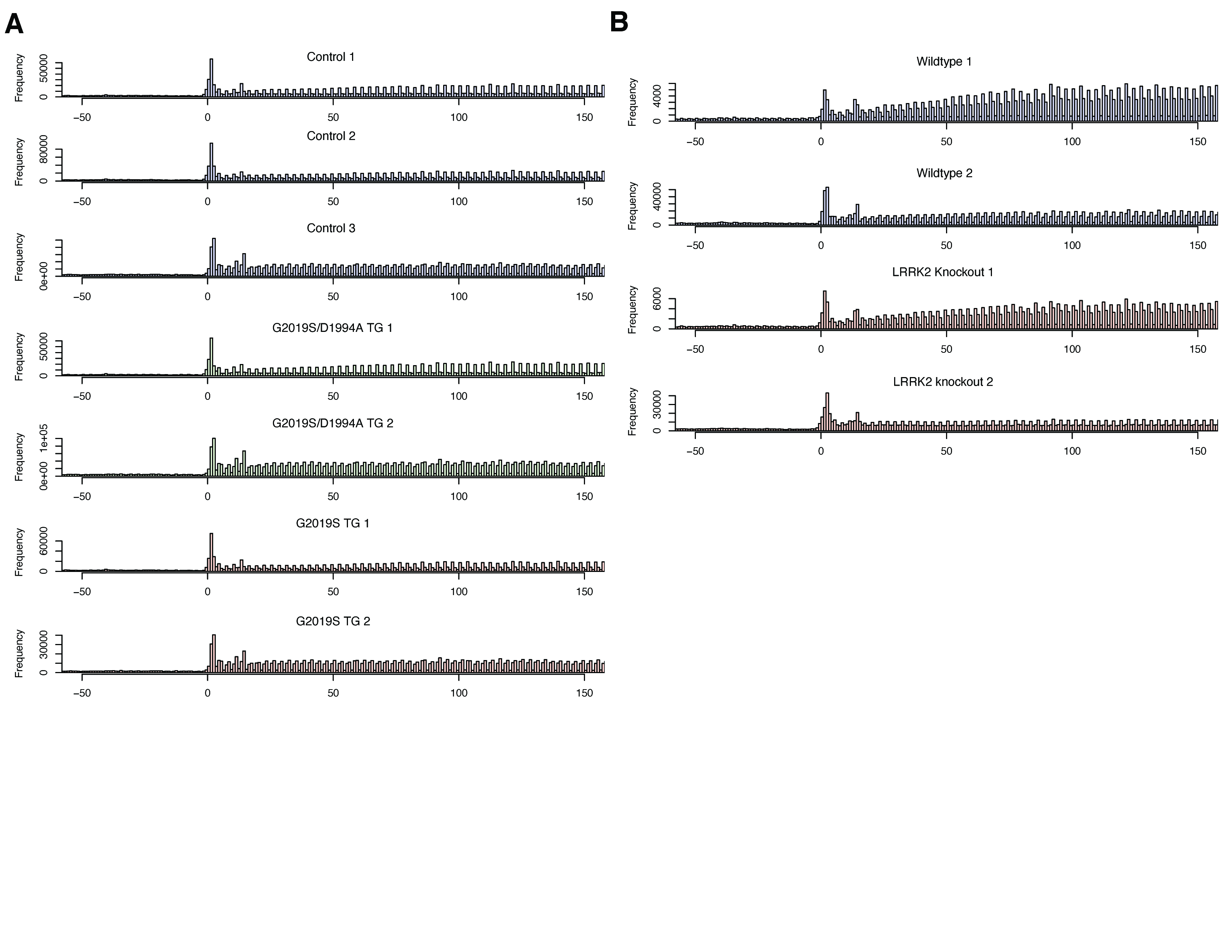

Supplement: Extended Data Figure 1-1 — Triple periodicity of ribosome profiling data. A, B, Triplet periodicity of ribosome profiling datasets were visualized to ensure the quality of the libraries. Transcript coordinates were re-aligned based on the rounded half point of the ribosome footprint [5′ end + (footprint length/2)]. Conserved triplet periodicity indicates that the libraries are faithfully representing translating ribosomes, ensuring the quality of the RPF libraries. There was no significant change found in ribosome footprint length, periodicity, and distribution in any LRRK2 mouse models (data not shown). Download Figure 1-1, TIF file. [file enu-eN-NWR-0310-21-s07.tif]

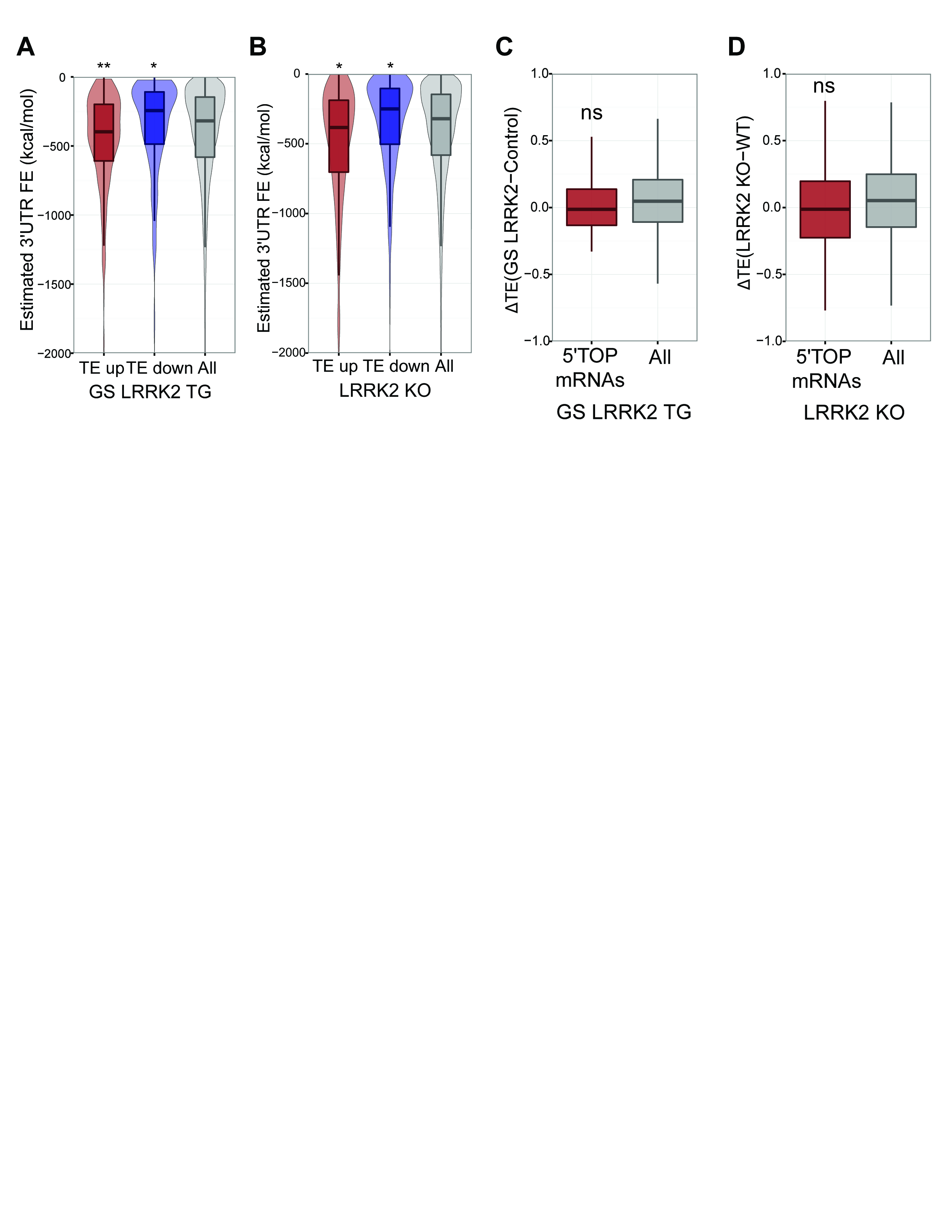

Supplement: Extended Data Figure 2-1 — 3′UTR secondary structure is not related to translational effects of G2019S LRRK2. A, B, 3′UTR secondary structure folding energy differences between TE up and TE down genes (standard z score ±1.5 was used). Unlike the 5′UTR folding energy comparison, 3′UTR folding energy did not show opposing directions of effects between G2019S (GS) LRRK2 transgenic (TG) and LRRK2 KO mice. Statistical significance was determined using Wilcoxon signed-rank test [A, p = 0.002338 (TE up), p = 0.02327 (TE down); B, p = 0.01194 (TE up), p = 0.0254 (TE down)]. C, D, TE differences of 5′ TOP mRNAs in LRRK2 mouse models. Wilcoxon signed-rank test; C, p = 0.2112; D, p = 0.09034. background signal. Error bars indicate SEM, *p < 0.05, **p < 0.01, ns = no significance. Download Figure 2-1, TIF file. [file enu-eN-NWR-0310-21-s04.tif]

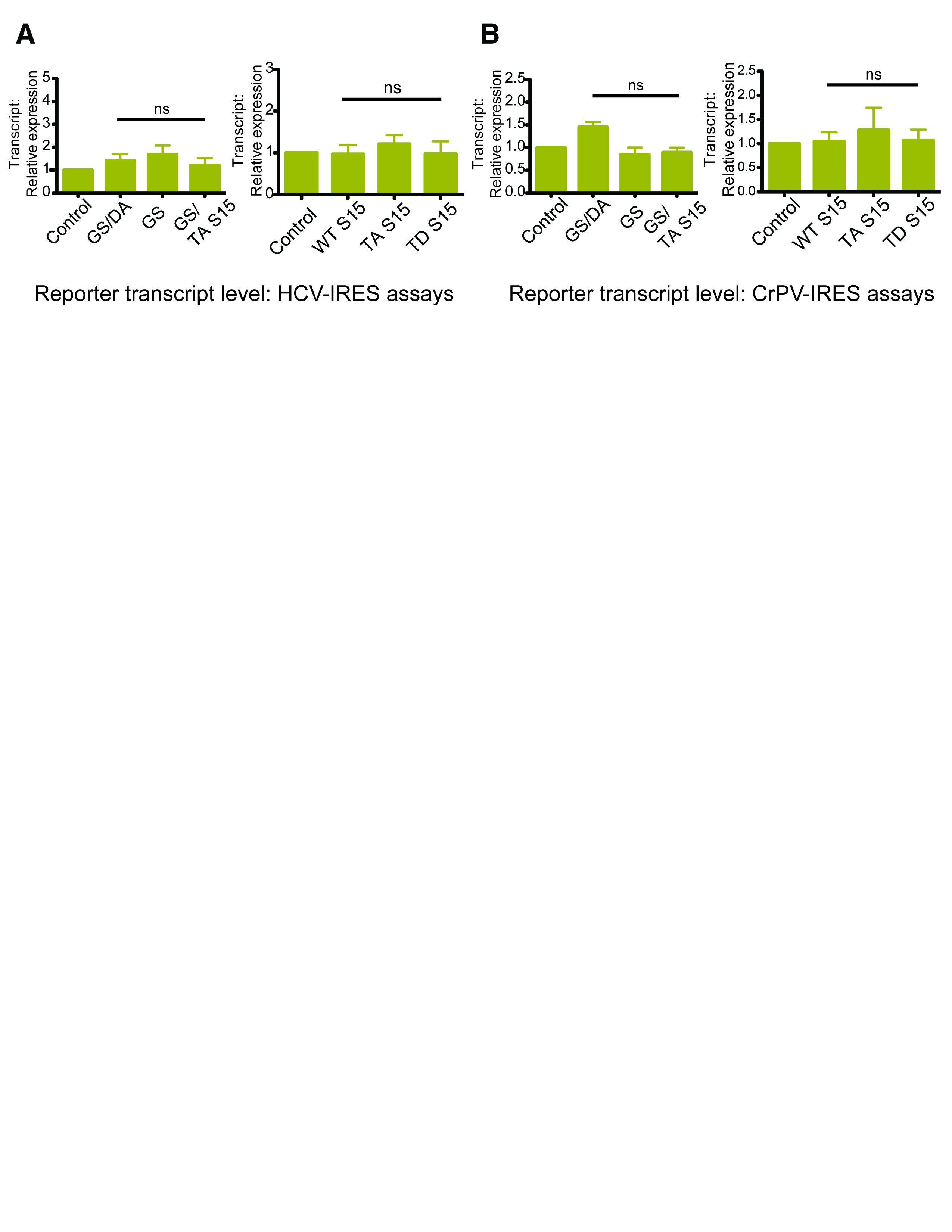

Supplement: Extended Data Figure 3-1 — Reporter transcript levels for IRES reporter assays. A, B, qPCR measurement of luciferase transcript levels in IRES reporter assays. One-way ANOVA with Bonferroni correction was used, and there were no significant changes in the reporter transcript levels detected; **p < 0.01, ***p < 0.001, ns = no significance. Download Figure 3-1, TIF file. [file enu-eN-NWR-0310-21-s05.tif]

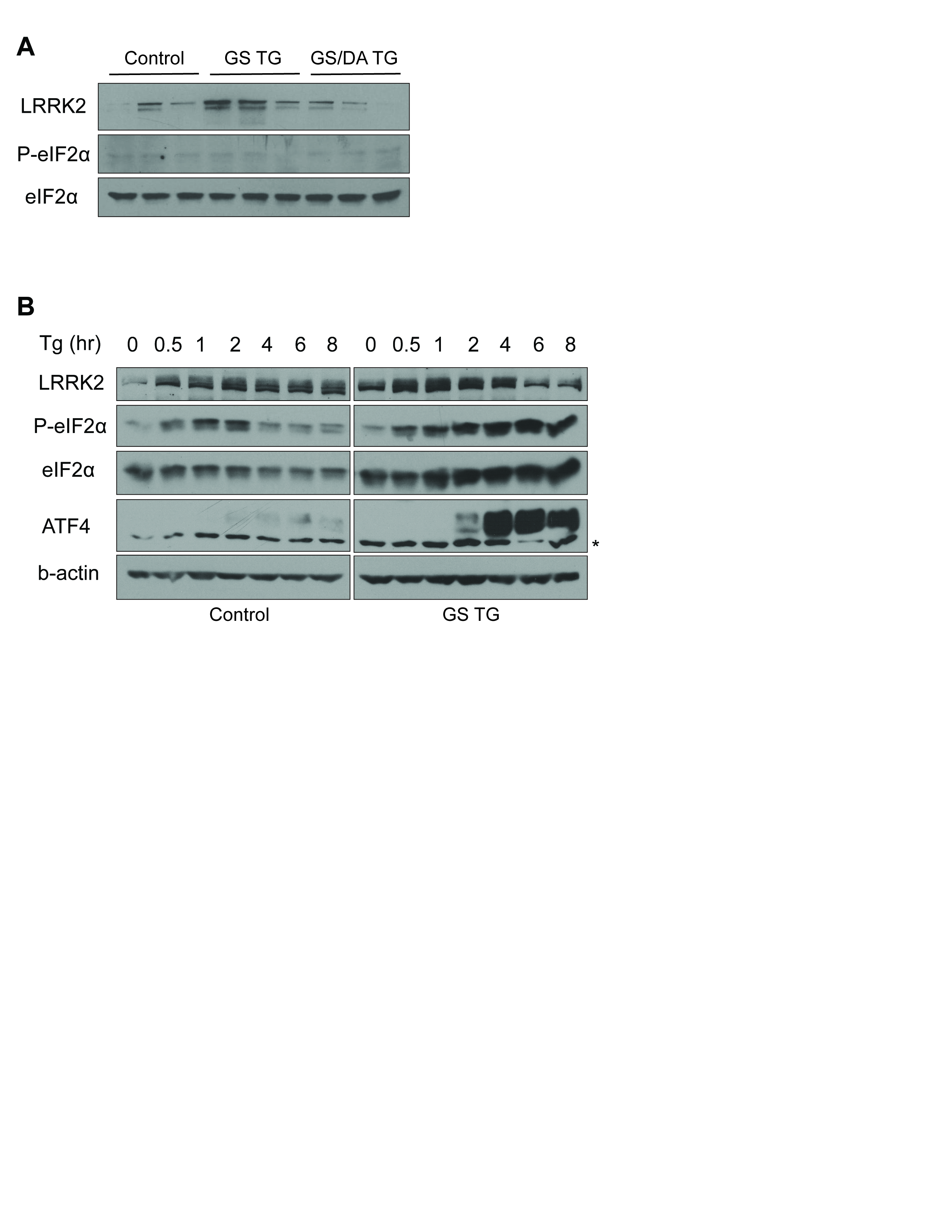

Supplement: Extended Data Figure 4-1 — Delayed ISR recovery from G2019S LRRK2-expressing neurons. A, Phosphorylation of eIF2ɑ in the G2019S LRRK2 transgenic brains. Dissected striatal tissues, age three to four months, n = 3, biological replicates. B, Mouse cortical neurons were prepared from pregnant transgenic breeders at E15. Pups were separated and individually genotyped. Control: wild type or single transgenic (CaMKII-tTA or tet-G2019S LRRK2), G2019S LRRK2: double transgenic. Tg: thapsigargin (1 μm). *, background signal. Download Figure 4-1, TIF file. [file enu-eN-NWR-0310-21-s06.tif]

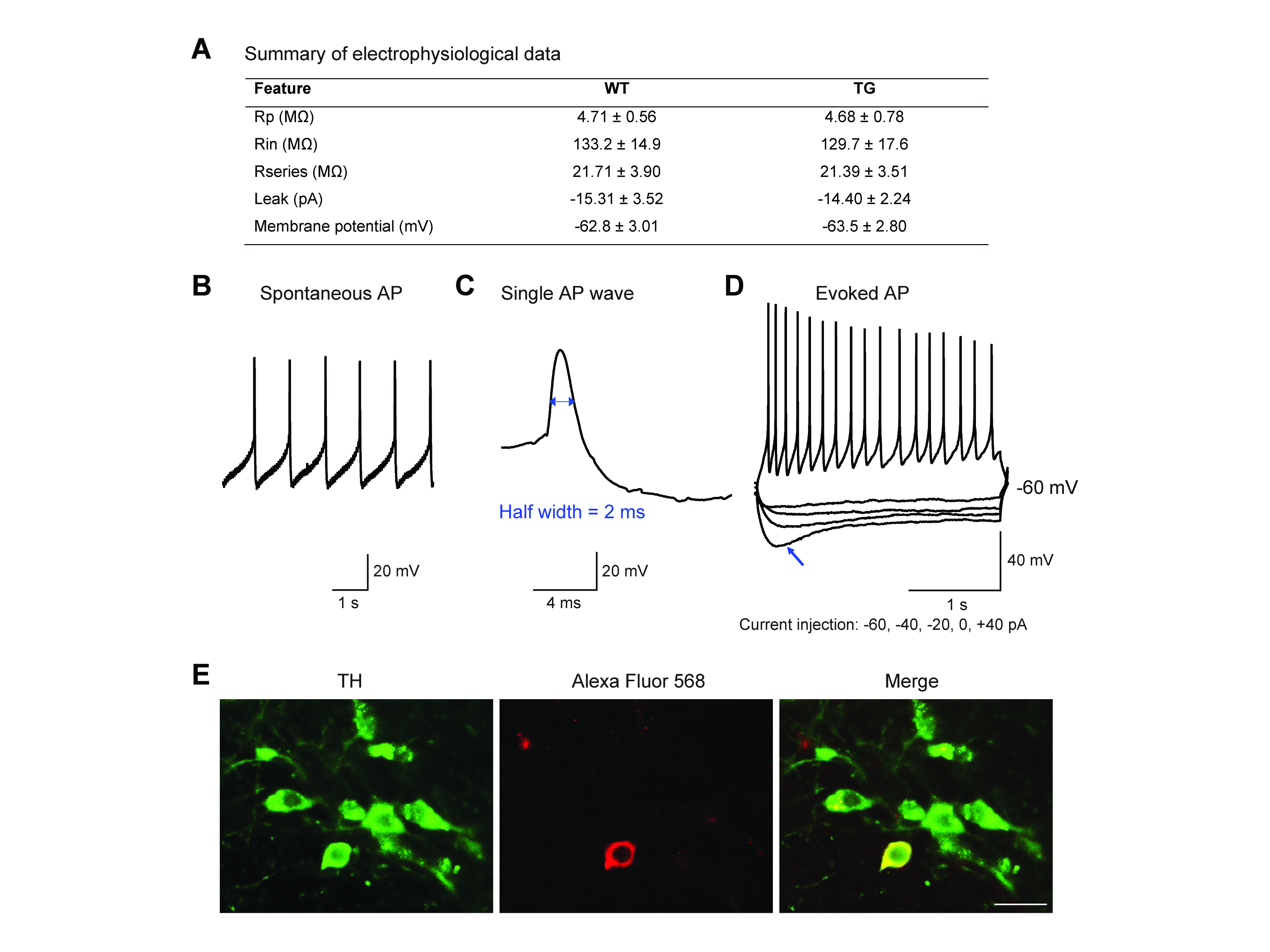

Supplement: Extended Data Figure 5-1 — Intrinsic properties of mouse brain DA neurons. A, Summary of electrophysiological characteristics of DA neurons in SNpc during recordings, including pipette resistance (Rp), input resistance (Rin), series resistance (Rseries), leak currents (Leak), and resting membrane potential. B, Spontaneous AP firing pattern in DA neurons. C, A representative single AP wave with a half width of 2 ms. D, Evoked APs. The presence of a sag (arrow) in the membrane potential and APs were detected in current-clamp immediately after rupturing the membrane. E, Immunofluorescence image showing recorded neurons are TH-positive. Alexa Fluor 568 was injected to label recorded neurons. Scale bar: 50 μm. Data are expressed as means ± SEM, WT, n = 6 slices from 6 mice; GS LRRK2, n = 6 slices from 6 mice. Download Figure 5-1, TIF file. [file enu-eN-NWR-0310-21-s08.tif]
